# Be Careful, Mom and Doc: Hepatotoxicity Associated with Prescribed Medications in Young Infants

**DOI:** 10.1155/2009/673269

**Published:** 2009-04-14

**Authors:** Kam-Lun Ellis Hon, Alexander K. C. Leung

**Affiliations:** ^1^Department of Pediatrics, The Chinese University of Hong Kong, Prince of Wales Hospital, Shatin, Hong Kong; ^2^Department of Pediatrics, Alberta Children's Hospital, University of Calgary, Calgary, AB, Canada T2W 3N2

## Abstract

Accidental poisonings in young infants are relatively uncommon, and the careless caregiver is usually the culprit. We report two cases of hepatotoxicity due to prescribed medications. An infant was given 15 mL instead of 1.5 mL of paracetamol by his mother because she omitted the decimal point on the label of the drug bottle. The infant became symptomatic, and liver enzyme and clotting profile were abnormal, necessitating treatment with N-acetyl cysteine. Another infant was prescribed oral ketoconazole for thrush, resulting in elevation of liver enzymes. The serum alanine aminotransferase levels were transiently elevated but returned to normal, and both infants recovered uneventfully. This report serves to alert the doctor to avoid using decimal points in drug labeling and to avoid prescribing excessive amount of drug for trivial acute illness. Thrush in infancy is common and usually treated with oral nystatin. Other oral antifungals such as ketoconazole may be associated with liver derangement and should be avoided in infants.

## 1. Introduction

Childhood poisonings are relatively uncommon and trivial in the metropolitan and densely populated city of Hong Kong [1]. Nevertheless, severe poisoning resulting in paediatric intensive care unit (PICU) admissions may occur [2]. We recently reported a 10-year survey of childhood poisoning and a 6-year survey of severe poisoning associated with PICU admissions [1, 2]. Accidental poisonings in infants are relatively uncommon but deserve mentioning. Unlike accidental ingestion due to curiosity in an older child, the poison is usually given by the caregivers in the case of a young infant, and the mother is usually the culprit. We reviewed clinical records of infants with prescribed medications associated with deranged liver function to illustrate the mechanism underlying the occurrence of such events.

## 2. Case Reports

### 2.1. Case 1

A 5-month-old 8.16 kg infant was prescribed paracetamol by a doctor for fever. The mother gave 15 mL instead of 1.5 mL of paracetamol three times a day because she omitted the decimal point on the label of the drug bottle. Three doses of 15 mL (containing 160 mg/5 mL of paracetamol) were given at 2.5 and 11 hours apart, respectively. The estimated total amount of paracetamol given was 1440 mg. The child developed vomiting, diarrhea, and irritability and was taken to the emergency department for assessment. The serum paracetamol was 70 *μ*mol/L at 14 hours following the last dose of paracetamol. The serum alanine aminotransferase was 115 IU/L (normal <58 IU/L) on admission and 292 IU/L 20 hours later. The clotting profile was also deranged with international ratio (INR) of 1.35 (normal: 0.9–1.1) and activated partial thromboplastin time (APTT) 51.9 seconds (normal: 26.2–40.1 seconds). He was given a full course of 18 doses of oral N-acetyl cysteine (1 gram initially followed by 570 mg every 4 hours) and the serum alanine aminotransferase and clotting profile gradually normalized.

### 2.2. Case 2

A 2-week-old girl born at 36-week gestation was diagnosed as having oral thrush. She was prescribed oral nystatin (1 mL 4 times per day) for 5 days which resulted in transiently improvement of the thrush. Nevertheless, the thrush recurred from time to time despite that the dosage of nystatin was increased stepwise to 1.5 mL and finally to 2 mL. At 4 months of age, the mother took the infant to see another doctor who prescribed a course of ketoconazole (Nizoral, Janssen Pharmaceutica, Belgium). She did not keep the bottles of these medications, and the exact sequence of events and details of dosages of nystatin and ketoconazole were not available. It was presumed that nystatin suspension was 1:100 000 U/mL and ketoconazole was 50 mg daily for 7 days. The doctor was concerned about possible hepatotoxicity, and liver function tests were ordered on completion of the ketoconazole which revealed that the serum alanine aminotransferase was elevated to 169 U/L (normal: 5–25 U/L) and aspartate aminotransferase to 128 U/L (normal: 15–60 U/L). The baby was asymptomatic and ketoconazole was discontinued. Consequently, the infant was referred to a teaching hospital for assessment. On examination, she was not jaundiced; the abdomen was soft and the oral cavity clear. Repeated liver function tests one week later showed that the serum alanine aminotransferase and aspartate aminotransferase had returned to normal, and the mother was reassured.

## 3. Discussion

Childhood poisoning is an important problem in many countries [3–9] and accounts for a significant workload for emergency department consultations and hospital admissions [10, 11]. These two cases illustrate hepatotoxicity associated with the treatment of two common conditions in infancy. In both cases, the mothers rightly took their children to their doctors for assessment and treatment. Nevertheless, hepatotoxicity developed as a consequence of inappropriate prescriptions. We derive a few general learning points from these cases.

**Point One**

Avoid using decimal point [12, 13]. This can be achieved by rounding the dosage up or diluting the dosage to an integral volume. Concomitantly, the doctor should prescribe a smaller total volume. Alternatively, a standard fixed-volume teaspoon may be used. A two-day prescription of 1.5 mL 3-times-per-day dosing would be 9 mL. It would have been virtually impossible for the mother to give one single 15 mL dose to the baby. Instead of asking for larger volume of prescription, the mother should be educated to take the child back for reassessment if fever does not resolve within 2 days. Often, paracetamol can be bought over the counter. As such, this suggestion may not work. Wrong dosage due to omitting the decimal points has been described [12, 13]. The doctors should avoid prescribing dosages with decimal points.

**Point Two**

Educate the mother about the pattern of fever associated with upper respiratory tract infection. The fever may last for a few days and may be worse in the evening. Paracetamol is the most common medication responsible for childhood ingestion and poisoning [1]. The half life of paracetamol is approximately 4–6 hours, and it may take 30 minutes for the onset of action. In our experience, many parents would give additional doses of the same prescription or prescriptions from different doctors or obtained over-the-counter albeit containing the same antipyretic ingredient. Some parents may even add rectal suppositories (also containing paracetamol) in between the oral doses. Paracetamol may accumulate after repeated therapeutic doses in children with fever [14]. Parents should be informed that the infant may not need the paracetamol regularly if the fever is low grade and the child is not bothered by it [14].

**Point Three**

Do not overwrap the infant. Tepid sponging with warm water may also help bring down the fever of a febrile child. This advice may be especially useful when antipyretic has just been given and the onset of effect is not immediately appreciable by the anxious caregiver.

**Point Four**

Many antifungal agents are excreted by the hepatic system and may be hepatotoxic. Thrush is a very common disease of infancy and usually takes one to two weeks of oral nystatin suspension to clear. As nystatin is not absorbed from the gut, it is safe for oral use and does not have problems of drug interactions. It is unnecessary to increase the dosages of an antifungal in quick successions. Potent antifungal agents such as ketoconazole are generally not indicated for thrush, and hepatic function must be monitored before and following their usage [15].

In conclusion, this report serves to alert the physicians to avoid using decimal points in drug labelling or prescribing excessive amount of medications for trivial acute illness. Thrush in infancy is common and usually treated with oral nystatin. Other oral antifungals such as ketoconazole may be associated with liver derangement and should be avoided in infants.
